# Adherence and Satisfaction of Smartphone- and Smartwatch-Based Remote Active Testing and Passive Monitoring in People With Multiple Sclerosis: Nonrandomized Interventional Feasibility Study

**DOI:** 10.2196/14863

**Published:** 2019-08-30

**Authors:** Luciana Midaglia, Patricia Mulero, Xavier Montalban, Jennifer Graves, Stephen L Hauser, Laura Julian, Michael Baker, Jan Schadrack, Christian Gossens, Alf Scotland, Florian Lipsmeier, Johan van Beek, Corrado Bernasconi, Shibeshih Belachew, Michael Lindemann

**Affiliations:** 1 Department of Neurology-Neuroimmunology Multiple Sclerosis Centre of Catalonia Vall d’Hebron University Hospital Barcelona Spain; 2 Department of Medicine Autonomous University of Barcelona Barcelona Spain; 3 Division of Neurology University of Toronto Toronto, ON Canada; 4 Department of Neurology University of California, San Diego San Diego, CA United States; 5 Department of Neurology University of California, San Francisco San Francisco, CA United States; 6 Genentech Inc South San Francisco, CA United States; 7 F Hoffmann–La Roche Ltd Basel Switzerland; 8 Department of Economics Baden-Wuerttemberg Cooperative State University Loerrach Germany

**Keywords:** multiple sclerosis, patient adherence, patient satisfaction, smartphone, wearable electronic devices, mobile phone

## Abstract

**Background:**

Current clinical assessments of people with multiple sclerosis are episodic and may miss critical features of functional fluctuations between visits.

**Objective:**

The goal of the research was to assess the feasibility of remote active testing and passive monitoring using smartphones and smartwatch technology in people with multiple sclerosis with respect to adherence and satisfaction with the FLOODLIGHT test battery.

**Methods:**

People with multiple sclerosis (aged 20 to 57 years; Expanded Disability Status Scale 0-5.5; n=76) and healthy controls (n=25) performed the FLOODLIGHT test battery, comprising active tests (daily, weekly, every two weeks, or on demand) and passive monitoring (sensor-based gait and mobility) for 24 weeks using a smartphone and smartwatch. The primary analysis assessed adherence (proportion of weeks with at least 3 days of completed testing and 4 hours per day passive monitoring) and questionnaire-based satisfaction. In-clinic assessments (clinical and magnetic resonance imaging) were performed.

**Results:**

People with multiple sclerosis showed 70% (16.68/24 weeks) adherence to active tests and 79% (18.89/24 weeks) to passive monitoring; satisfaction score was on average 73.7 out of 100. Neither adherence nor satisfaction was associated with specific population characteristics. Test-battery assessments had an at least acceptable impact on daily activities in over 80% (61/72) of people with multiple sclerosis.

**Conclusions:**

People with multiple sclerosis were engaged and satisfied with the FLOODLIGHT test battery. FLOODLIGHT sensor-based measures may enable continuous assessment of multiple sclerosis disease in clinical trials and real-world settings.

**Trial Registration:**

ClinicalTrials.gov: NCT02952911; https://clinicaltrials.gov/ct2/show/NCT02952911

## Introduction

Disease progression throughout the clinical course of multiple sclerosis (MS) is measured using clinician-reported outcomes, most commonly the Expanded Disability Status Scale (EDSS) [1], magnetic resonance imaging (MRI), and patient-reported outcomes (PROs). Conventional assessment of the clinical course of MS relies on relapse-associated and periodic in-clinic visits. However, the current intermittently conducted clinic-based outcome measures in MS have limitations in studying the insidiously subtle progression in MS and may fail to comprehensively capture transient symptomatic and performance fluctuations that affect people with multiple sclerosis.

The ability of consumer wearable technology to measure functional impairment associated with various neurological disease symptoms through smartphone-based assessments is an important area of research [2-5]. Smartphones as vehicles for sensor-based technologies offer the potential for enhanced active and passive real-time data capture that may fundamentally shift traditional paradigms of clinical monitoring [6-9]. A recent large-scale study demonstrated that more than 95% of people with multiple sclerosis have access to a mobile device and most use it routinely [10]. Recently published studies have described the use of technologies in developing tools to assess people with multiple sclerosis [7,9,11-13]. In a recent study, it was reported that both healthy participants and people with multiple sclerosis were capable of completing daily tasks on a smartphone for 1 year [7]. Collection of data on a variety of cognitive and motor tests via the smartphone may represent a feasible way to gather highly granular data to accurately describe the MS disease course outside of the clinic [7].

The FLOODLIGHT study [NCT02952911] was a prospective pilot study to assess the feasibility of remote measurements using smartphones and smartwatches in people with multiple sclerosis and healthy controls (HCs). The smartwatch and smartphone contained apps that prompted the user to perform various assessments and protocols, referred to as active tests. The app also passively recorded sensor data during daily life, referred to as passive monitoring. The novel smartphone- and smartwatch-based FLOODLIGHT active tests were developed to be self-administered by people with multiple sclerosis to capture MS symptoms, including hand motor function, gait and posture, mood, and cognitive impairment.

The primary objectives of the FLOODLIGHT study, which began in November 2016, were to evaluate participant adherence to smartphone- and smartwatch-based assessments and collect feedback from people with multiple sclerosis and HCs on the smartphone and smartwatch schedule of assessments and its impact on their daily activities using a patient satisfaction questionnaire. Other objectives of the study, which will be addressed in subsequent publications, are to determine the association between exploratory sensor-based outcomes derived from the respective components of the FLOODLIGHT test battery and conventional MS clinical outcomes and explore whether the FLOODLIGHT test battery can differentiate between participants with and without MS.

## Methods

### Trial Design and Participants

After providing written informed consent, people with multiple sclerosis and HCs, preferentially partners or cohabitants, were evaluated for eligibility for enrollment in the FLOODLIGHT study. Eligibility criteria for people with multiple sclerosis included the ability to comply with the study protocol, age 18 to 55 years, diagnosis of MS (2010 revised McDonald criteria, treated or untreated) [14], EDSS score 0 to 5.5 (inclusive), and weight from 99 to 243 lbs (45 to 110 kg). An EDSS score of 5.5 as a maximum limit was meant to ensure any patient with relapsing or progressive MS would not have any significant difficulty in participating in the proposed testing as per study protocol. Further details on eligibility criteria are provided in Multimedia Appendix 1.

This study was conducted at two sites in two countries with a total of 76 people with multiple sclerosis and 25 HCs; 60 people with multiple sclerosis and 20 HCs were recruited from the Multiple Sclerosis Centre of Catalonia, Vall d’Hebron University Hospital, Barcelona, Spain, and 16 people with multiple sclerosis and 5 HCs were recruited from the University of California, San Francisco, California.

The protocol, informed consent forms, any information given to the participants, and relevant supporting information were reviewed and approved by the institutional review board/ethics committee before the study was initiated. Confidentiality was maintained by assigning each participant enrolled in the study a unique identification number.

### Study Design

**Figure 1:**
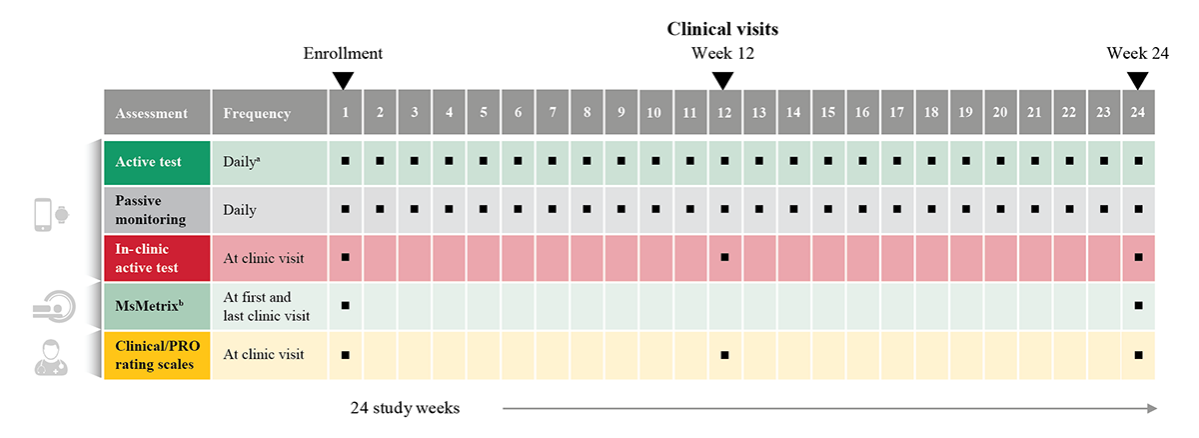
FLOODLIGHT study design. PRO: patient-reported outcome. "a" indicates that active tests were administered weekly or every two weeks (see next figure for schedule). "b" indicates that brain magnetic resonance imaging was performed in people with multiple sclerosis.

The FLOODLIGHT study combines continuous sensor data capture with smartphones and smartwatches and standard clinical outcome measures. Eligible people with multiple sclerosis and HCs were enrolled in the study and assessed clinically at the enrollment visit, week 12, and termination visit (week 24). In addition, participants were asked to perform a set of daily active tests and contribute sensor data via passive monitoring with smartphone and smartwatches over a period of 24 weeks (Figure 1).

### In-Clinic Assessments

At each scheduled in-clinic visit (enrollment, week 12, and week 24), the following reference clinical tests were performed for all participants: 9-Hole Peg Test (9HPT), oral version of Symbol Digit Modalities Test (SDMT) [15-17], Timed 25-Foot Walk (T25FW) test, Berg Balance Scale (BBS) [18], Fatigue Scale for Motor and Cognitive Functions (FSMC) [19], and Patient Health Questionnaire–9 (PHQ-9) [20]. For people with multiple sclerosis only, disability was measured by EDSS [1], Patient Determined Disease Steps (PDDS) [21], and Multiple Sclerosis Impact Scale–29 (MSIS-29; version 2) [22-24]. While performing some of the in-clinic tests, people with multiple sclerosis and HCs were asked to carry or wear the smartphone and smartwatch to collect sensor data alongside the in-clinic measures. On the scheduled in-clinic visits, the smartphone and smartwatch FLOODLIGHT active tests were performed under investigator supervision. The satisfaction questionnaire (Multimedia Appendix 2) assessed people with multiple sclerosis’ and HCs’ experience regarding smartphone and smartwatch use and its impact on their daily activities at the week 12 visit and at the study termination/early discontinuation visit. Brain MRI was performed in people with multiple sclerosis at the enrollment visit and at week 24.

### Smartphone and Smartwatch Testing

At the enrollment visit, people with multiple sclerosis and HCs were provided with the FLOODLIGHT solution that included a smartphone and smartwatch preconfigured so participants could only run the FLOODLIGHT software. A belt bag was also provided for participants to carry their smartphone in an anterior medial position. The smartphone and smartwatch pair contained preinstalled apps that prompted the user to perform various assessments, referred to as active tests. The apps also passively recorded sensor data, referred to as passive monitoring. At the enrollment visit, participants received training on the use of the smartphone and smartwatch and were provided with supporting content to help them complete the tests successfully. Participants were instructed to complete the active tests at approximately the same time each day and carry the smartphone and smartwatch throughout the day, recharging the devices overnight. Data transfer from the smartphone and smartwatch is described in Multimedia Appendix 1.

### FLOODLIGHT Active Tests

**Figure 2:**
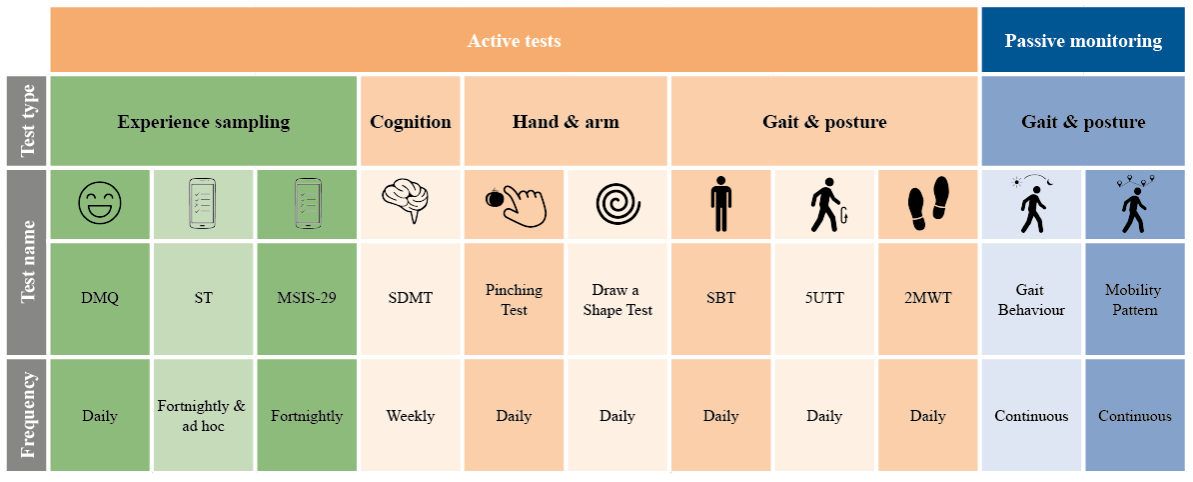
FLOODLIGHT active tests and their schedule frequency. DMQ: Daily Mood Question; MSIS-29: Multiple Sclerosis Impact Scale–29; SBT: Static Balance Test; SDMT: Symbol Digit Modalities Test; ST: Symptom Tracker; 2MWT: Two-Minute Walk Test; 5UTT: 5 U-Turn Test.

People with multiple sclerosis and HCs were asked to perform various active tests (daily, weekly, every two weeks, or on demand) via the smartphone (Figure 2 and Table 1). These novel active tests were developed to be self-administered by people with multiple sclerosis to capture MS symptoms. A range of clinical and sensor-based assessments were chosen to capture the most prominent symptoms of MS from a broad spectrum of symptoms. People with multiple sclerosis and HCs were required to wear the smartwatch throughout the active tests.

**Table 1:** FLOODLIGHT active tests.

Domain and test		Short description
**Daily hand motor function tests^a^**		
	Draw a Shape (DaS) Test	The aim of the DaS Test is to assess fine finger/manual dexterity while the participants are instructed to hold the mobile device in the untested hand and draw on the smartphone touchscreen six prewritten alternating shapes of increasing complexity (linear, rectangular, circular, sinusoidal, and spiral) with the second finger of the tested hand as fast and as accurately as possible within a maximum time (30 seconds for each of the two attempts per shape).
	Pinching Test	The aim of the Pinching Test is to assess fine pinching/grasping dexterity while the participants are instructed to hold the mobile device in the untested hand and touch the screen with two fingers from the tested hand (thumb + second or thumb + third finger preferred) to squeeze/pinch as many round shapes (ie, tomatoes) as they can during 30 seconds.
**Daily gait tests^b^**		
	Two-Minute Walk Test (2MWT)	Participants are instructed to walk as fast and as long as they can for 2 minutes but walk safely. The 2MWT is a simple test that is required to be performed on an even ground in a place where participants have identified they could walk straight for as far as ≥200 meters without U-turns. Participants are allowed to wear regular footwear and an assistive device and/or orthotic as needed.
	5 U-Turn Test (5UTT)	The aim of this test is to assess difficulties or unusual patterns in performing U-turns while walking on a short distance at comfortable pace. The 5UTT can be performed indoors or outdoors, on an even ground where participants are instructed to walk safely and perform five successive U-turns going back and forward between two points a few meters apart for 1 minute. Participants are allowed to wear regular footwear and an assistive device and/or orthotic as needed.
	Static Balance Test (SBT)	Participants are asked to stand still unsupported for 30 seconds with relaxed arms straight alongside the body if possible.
**Weekly cognitive test**		
	Electronic version of the Symbol Digit Modalities Test (SDMT) [15-17]	The aim of SDMT testing is to detect impairment of key neurocognitive functions that underlie many substitution tasks.
**Patient-reported outcomes (PROs)**		
	Daily Mood Question (DMQ)	This test represents an assessment of participants’ perceived overall state by responding daily to the question “How do you feel now?” on a 5-item Likert scale, ranging from excellent to horrible.
	Electronic version of the Multiple Sclerosis Impact Scale–29, version 2 (MSIS-29) [22-24]; people with multiple sclerosis only	This questionnaire measures the physical and psychological impact of multiple sclerosis.
	Multiple Sclerosis Symptom Tracker (MSST); people with multiple sclerosis only	Patients are asked if they experienced new or significantly worsening symptoms during the last 2 weeks. If yes, onset of the symptoms and the patients’ perception to whether they think they experienced a relapse (yes, no, or unsure) are recorded.

^a^Tests alternatingly performed with right and left hand; users are instructed on daily alternation.

^b^Recommended position of smartphone in an anterior medial position in the belt bag.

### FLOODLIGHT Passive Monitoring

Passive monitoring collected metrics on gait and mobility throughout the day in a continuous and unobtrusive manner. Participants were instructed to carry their smartphone preferably in an anterior medial position in a belt bag or, alternatively, in their pocket and wear the smartwatch all day as they went about their daily routine until the devices ran out of charge.

### Statistical Analyses

The analyses of the primary objectives of this study were descriptive. Statistical tests were exploratory and conducted at the two-sided 5% significance level without adjustment for multiple comparisons. The analyses were based on all enrolled patients (full analysis set [FAS]). Patients who prematurely withdrew from the study for any reason were still included in the FAS. Supportive analyses of selected variables were carried out in the per-protocol population, which included people with multiple sclerosis who completed at least 1 week in the study and did not discontinue due to an adverse event or a reason unrelated to the use of the FLOODLIGHT solution (Multimedia Appendix 3).

Adherence was evaluated for the following tests and test groups: all FLOODLIGHT active tests, Two-Minute Walk Test (2MWT), all active tests except 2MWT, smartphone use, smartwatch use, and for the per-protocol population.

Adherence to active tests was measured as the proportion of study weeks with at least 3 days of completed testing (study co-primary endpoint). Adherence to sensor-based passive monitoring was measured as the proportion of study weeks with at least 3 days of passive monitoring for at least 4 hours per day while the devices were worn by the participant [25] (study co-primary endpoint). Descriptive statistics of calculated adherence were reported for all active tests, all active tests except 2MWT, 2MWT, smartphone use, and smartwatch use. Categorical and numeric variables with fewer than five values were tested for association with adherence using the Kruskal-Wallis test. The association between continuous variables was assessed using the Spearman rank correlation.

Participant complete abandoning of active testing and passive monitoring was also investigated in a time-to-event survival analysis based on the Kaplan-Meier method along the FLOODLIGHT study. The abandoning event was defined as the last week in which the participant was adherent according to the definitions above for active tests and passive monitoring. Active tests performed on days of in-clinic visits were not considered in the adherence calculation to focus the abandoning analysis on the remote use. Participants leaving the study before the terminal visit were considered as censored. The impact of different characteristics on adherence was assessed using Cox regression.

A satisfaction score was developed from the satisfaction questionnaire (Multimedia Appendix 2) that sums the individual answers to questions 1-7 and 10-12 rescaled to 0-100 from their original Likert scale. An interquestion correlation analysis was performed to ensure questions are equally correlated and can be combined. Descriptive statistics of satisfaction score and items are reported, along with covariate analyses of demographics and disease state. Analysis of the change in satisfaction score between week 12 and week 24 are reported using the Wilcoxon signed-rank test.

Patient baseline characteristics incorporated as covariates in the analysis of correlation with FLOODLIGHT adherence and satisfaction outcomes were age, gender, body mass index, time since first MS symptom onset, EDSS, T25FW time, 9HPT time, and the oral SDMT correct responses. A descriptive analysis of safety variables, including adverse events and serious adverse events, was carried out in the FAS.

## Results

### Baseline Demographics and Characteristics

Participants’ baseline demographics for the FAS are described in Table 2. There was an expected greater proportion of females among the people with multiple sclerosis compared with HCs. The majority (69/76, 91%) of people with multiple sclerosis had relapsing-remitting MS (RRMS), with a mild EDSS score (mean 2.4) and presumably “normal” hand/arm function based on an upper limit of normal range defined as the average 9HPT time for HCs plus two standard deviations [26] (Table 2).

Overall, 92% (70/76) of people with multiple sclerosis and 64% (16/25) of HCs who enrolled in the FLOODLIGHT study reached the week 24 visit. Reasons for discontinuation from the study are described in Multimedia Appendix 3.

**Table 2:** Demographics and characteristics of people with multiple sclerosis and healthy controls (HCs) at baseline.

Parameter		People with multiple sclerosis (n=76)	HCs (n=25)
Age (years), mean (SD)		39.5 (7.9)	34.9 (9.3)
Female, n (%)		53 (70)	7 (28)
**Multiple sclerosis (MS)** **diagnosis, n (%)**			
	Primary progressive multiple sclerosis	3 (4)	—^a^
	Secondary progressive multiple sclerosis	4 (5)	—
	Relapsing-remitting multiple sclerosis	69 (91)	—
Time since MS symptom onset (years), mean (SD)		11.3 (7.0)^b^	—
Proportion of people with multiple sclerosis with ≥1 relapse in the past year, n (%)		18 (24)	—
Expanded Disability Status Scale, mean (SD)		2.4 (1.4)	—
Proportion of people with multiple sclerosis with ≥1 T1 Gd^c^-enhancing lesion, n (%)		2 (3)^d^	—
Total FLAIR^e^ lesion volume (mL), mean (SD)		6.3 (7.5)^f^	—
**9-Hole Peg Test** **(seconds), mean (SD)**			
	Dominant hand	22.1 (4.6)^g^	18.9 (2.1)
	Nondominant hand	22.8 (4.9)^h^	19.5 (2.0)
Timed 25-Foot Walk (seconds), mean (SD)		6.0 (2.1)^b^	5.0 (1.0)
Symbol Digit Modalities Test (correct responses), mean (SD)		53.8 (11.8)^b^	63.8 (10.0)
Berg Balance Scale, mean (SD)		52.5 (5.7)^i^	56.0 (0)^j^
Patient Determined Disease Steps, mean (SD)		1.5 (1.6)	—
Fatigue Scale for Motor and Cognitive Functions (total score), mean (SD)		59.1 (22.7)^g^	25.5 (6.0)
Patient Health Questionnaire–9, mean (SD)		8.3 (6.1)^k^	2.4 (2.9)^l^
Participants with any previous medications, n (%)		46 (61)	6 (24)
**Previous disease-modifying treatment^m^, n (%)**			
	Daclizumab (Zinbryta)	0 (0)	—
	Glatiramer acetate (Copaxone)	12 (16)	—
	Glatiramer acetate (Glatopa)	1 (1)	—
	IFN^n^ β-1a IM^o^ (Avonex)	4 (5)	—
	IFN β-1a SC^p^ (Rebif)	5 (7)	—
	IFN β-1b SC (Betaseron/Betaferon)	6 (8)	—
	IFN β-1b SC (Extavia)	1 (1)	—
	Pegylated IFN β-1a (Plegridy)	2 (3)	—
	Dimethyl fumarate (Tecfidera)	9 (12)	—
	Fingolimod (Gilenya)	9 (12)	—
	Teriflunomide (Aubagio)	3 (4)	—
	Alemtuzumab (Lemtrada)	2 (3)	—
	Mitoxantrone (Novantrone)	1 (1)	—
	Natalizumab (Tysabri)	19 (25)	—
	Other^q^	5 (7)	—

^a^Not applicable.

^b^n=75.

^c^Gd: gadolinium.

^d^n=68.

^e^FLAIR: fluid-attenuated inversion recovery.

^f^n=70.

^g^n=73.

^h^n=74.

^i^n=71.

^j^n=22.

^k^n=60.

^l^n=20.

^m^Total baseline disease-modifying treatment history.

^n^IFN: interferon.

^o^IM: intramuscular.

^p^SC: subcutaneous.

^q^Hidroferol; Radiance study (RPC1063 versus IFN β-1a); Rituximab (Rituxan).

### Adherence

**Figure 3:**
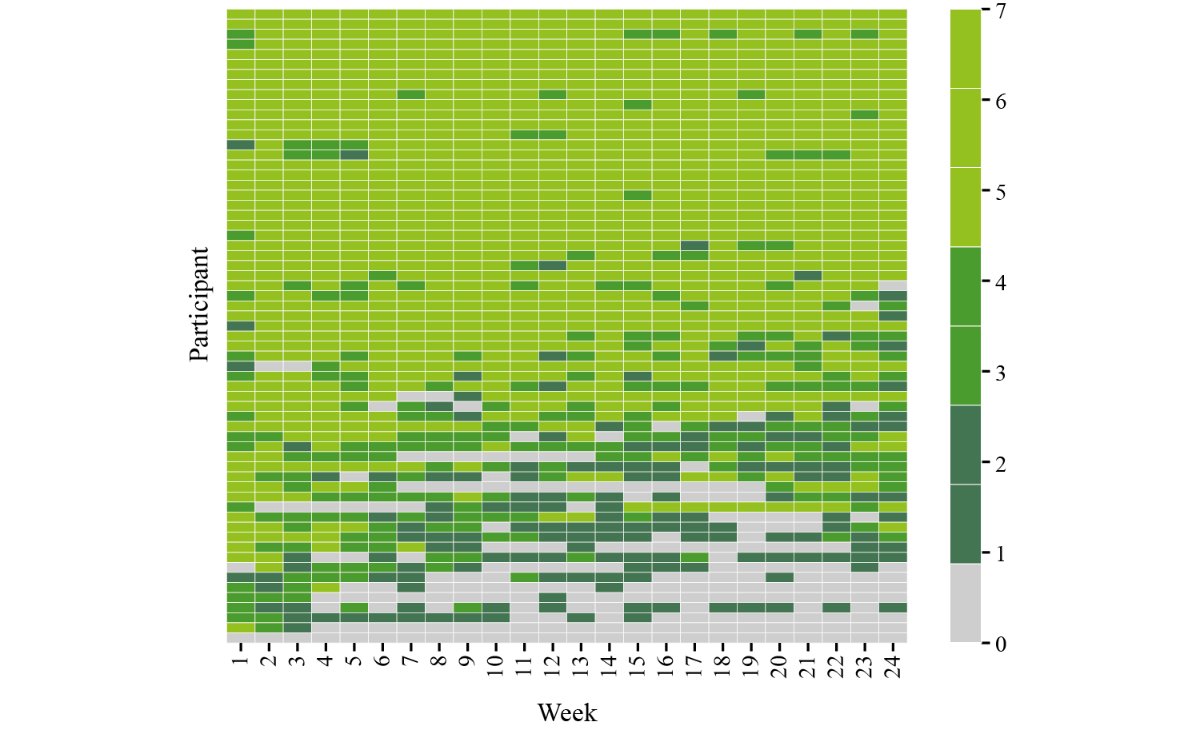
Adherence of people with multiple sclerosis to active tests for individual participants: number of performed active tests per week [level of activity (light green: high; dark green/grey: low] over individual study weeks [columns]).

**Figure 4:**
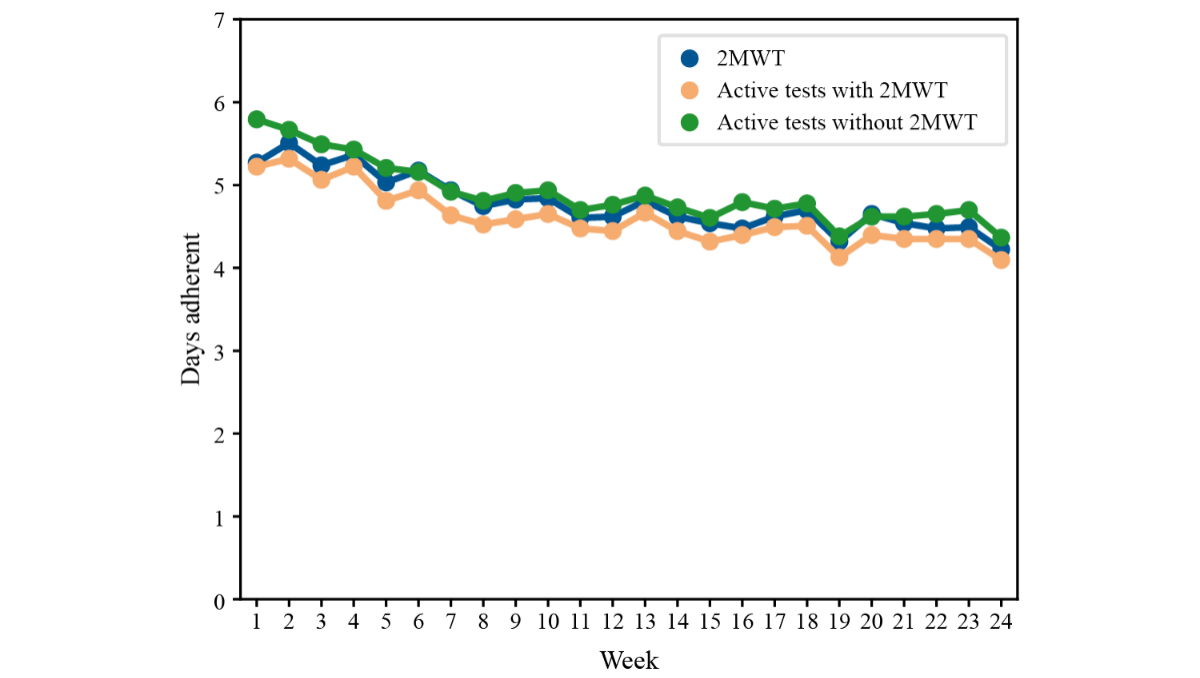
Adherence of people with multiple sclerosis to active tests. 2MWT: Two-Minute Walk Test.

**Figure 5:**
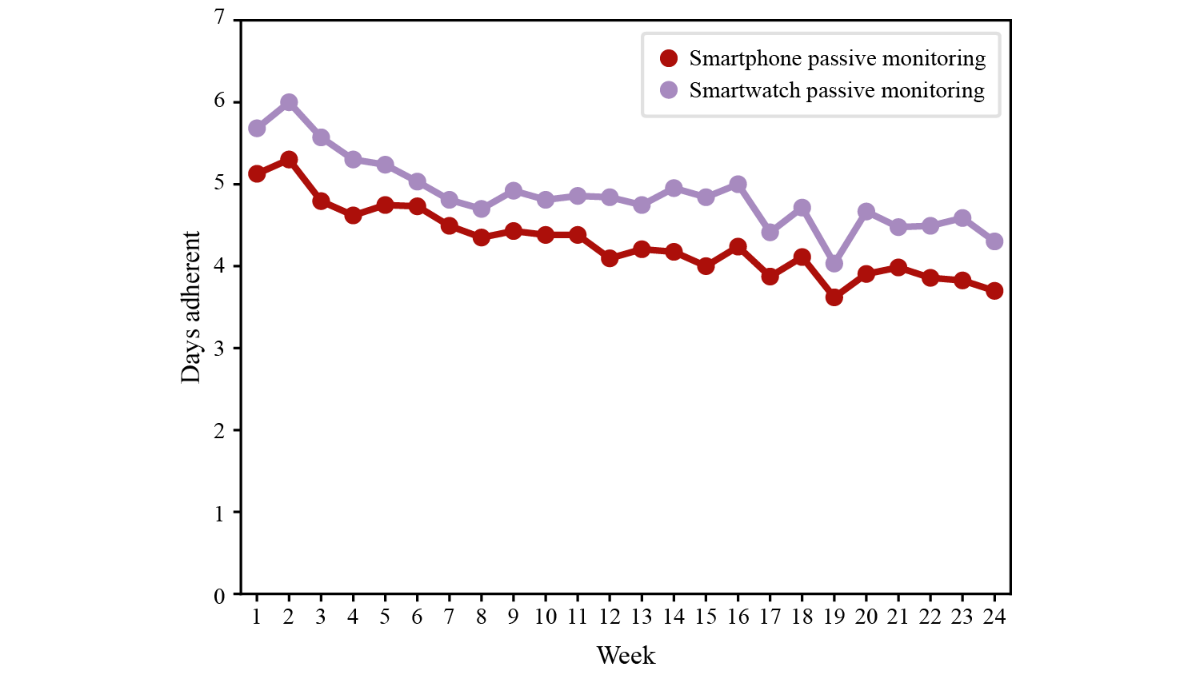
Adherence of people with multiple sclerosis to smartphone and smartwatch passive monitoring. Days with more than 4 hours of passive monitoring on a device are considered as adherent.

**Figure 6:**
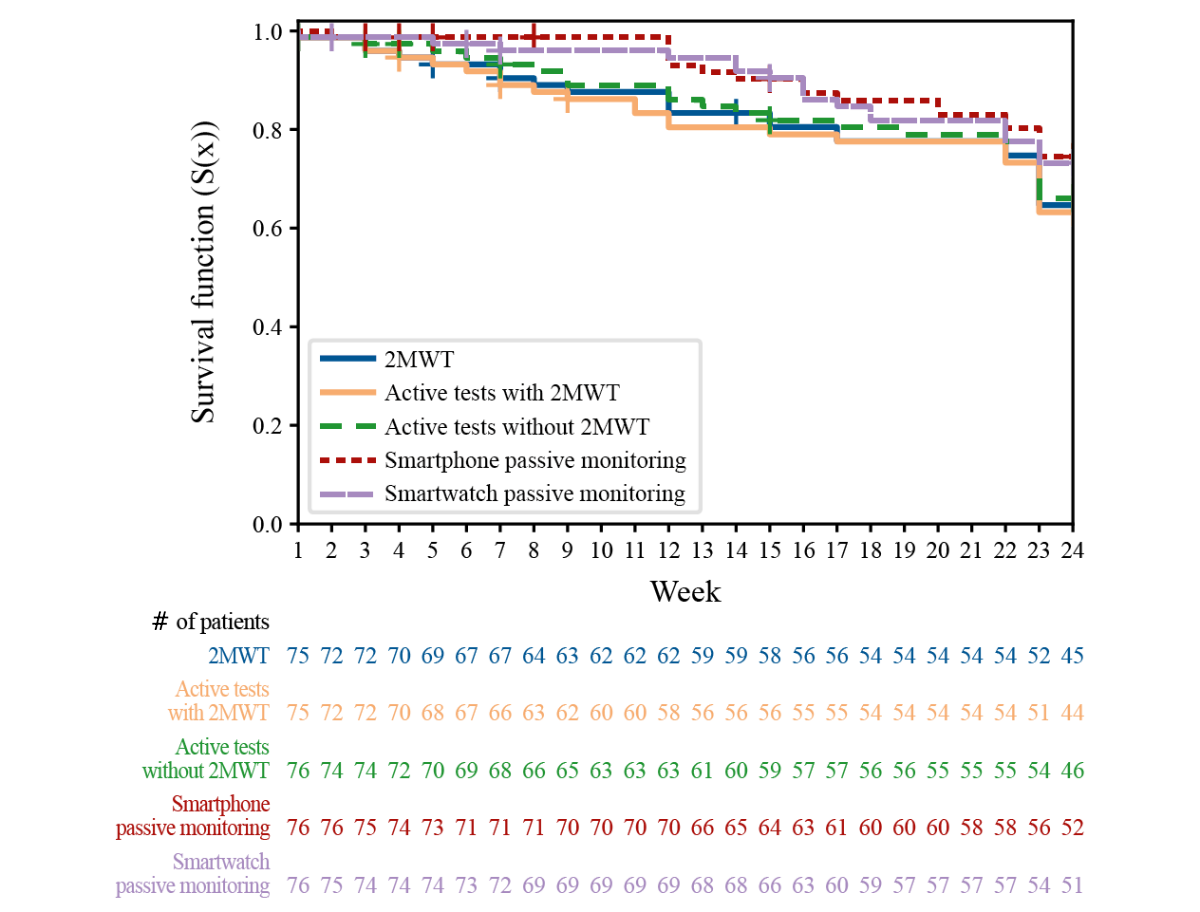
Adherence of people with multiple sclerosis to active tests and passive monitoring. The results of the time-to-event survival analysis based on the Kaplan–Meier method along the FLOODLIGHT study. The abandoning event was defined as the last week in which the participant was adherent according to the definitions for active tests and passive monitoring. Active tests performed on days of in-clinic visits were not considered in the adherence calculation, to focus the abandoning analysis on the remote use. Participants leaving the study before the terminal visit were considered as censored. 2MWT: Two-Minute Walk Test.

Over a period of 18 months (November 2016-April 2018), more than 6 terabytes of raw data were collected from 76 people with multiple sclerosis and 25 HCs. Participants performed 67,544 active tests, of which 9787 were the 2MWT, and recorded 200,171 hours of passive monitoring, of which 113,165 hours were captured with the smartwatch. Over 24 weeks, most participants performed 5 to 7 active tests per week, including the 2MWT (Figure 3). Adherence of people with multiple sclerosis to completing active tests and passive monitoring was good and remained stable over time after week 6 (Figures 4 and 5). Even in the last week of the 24-week study, participants completed all active tests on average 4 out of 7 days per week (Figure 4), and recorded at least 4 hours of data via passive monitoring on average 4 out of 7 days per week (Figure 5). The lowest average adherence over 24 weeks was observed for active tests including the 2MWT and the 2MWT only, with participants showing highest average adherence for passive monitoring (Figure 6). A total of 70% (16.68/24 weeks) of participants were adherent to all active tests, 75% (17.95/24 weeks) to all active tests except 2MWT, 71% (17.13/24 weeks) to 2MWT, 79% (18.89/24 weeks) to smartphone- or smartwatch-based passive monitoring, 66% (15.74/24 weeks) to smartphone-based passive monitoring, and 74% (17.69/24 weeks) to smartwatch-based passive monitoring.

Correlation was explored between adherence measures and people with multiple sclerosis population characteristics. Only disease duration showed significant small negative correlation with measures of adherence (Spearman rank correlations; 2MWT adherence: –0.42, *P*<.001; smartphone passive monitoring adherence: –0.29, *P*=.02; all active tests except 2MWT adherence: –0.37, *P*=.003; and smartwatch passive monitoring adherence: –0.27, *P*=.04), indicating that disease severity and demographics did not appear to play a significant role in adherence.

### Patient Satisfaction

The average overall satisfaction score among people with multiple sclerosis who completed the study at week 12 (n=64) was 74.1 out of a possible 100 and remained stable at week 24 (study termination/early discontinuation visit [n=68]) with 73.7 out of 100 (Wilcoxon signed-rank test *P*=.71). There was one significant association between overall satisfaction score and gender (*P*=.04). Individual questions from the satisfaction questionnaire (Multimedia Appendix 2) were analyzed for their association with people with multiple sclerosis population characteristics, described in Multimedia Appendix 1.

**Figure 7:**
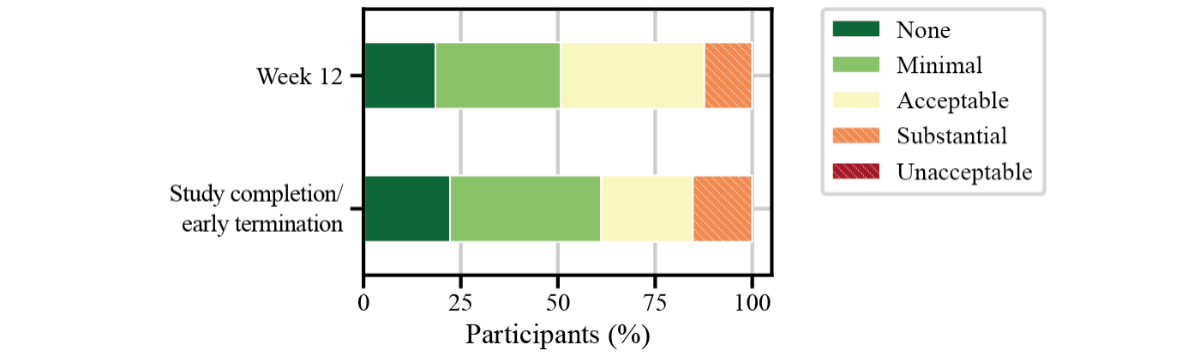
Implications of FLOODLIGHT in people with multiple sclerosis for “impact on daily activities” from the patient satisfaction questionnaire.

**Figure 8:**
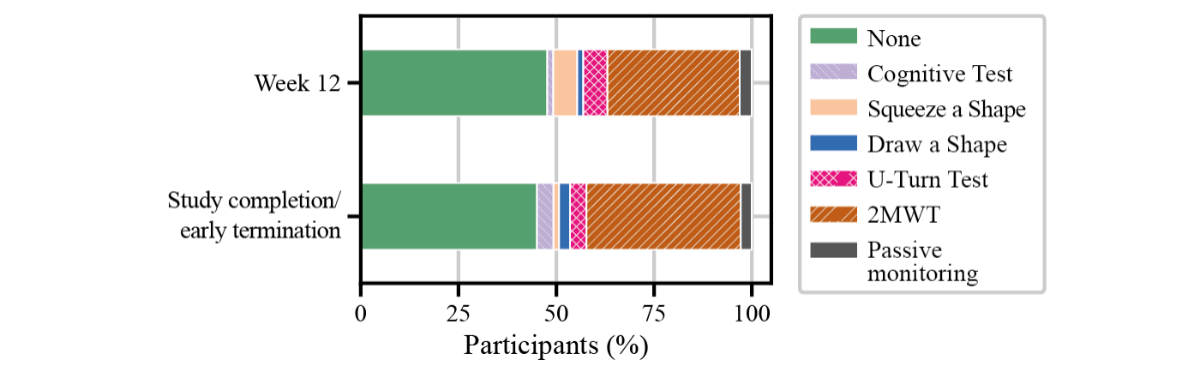
Implications of FLOODLIGHT in people with multiple sclerosis for “avoiding one component of FLOODLIGHT" from the patient satisfaction questionnaire. 2MWT: Two-Minute Walk Test.

**Figure 9:**
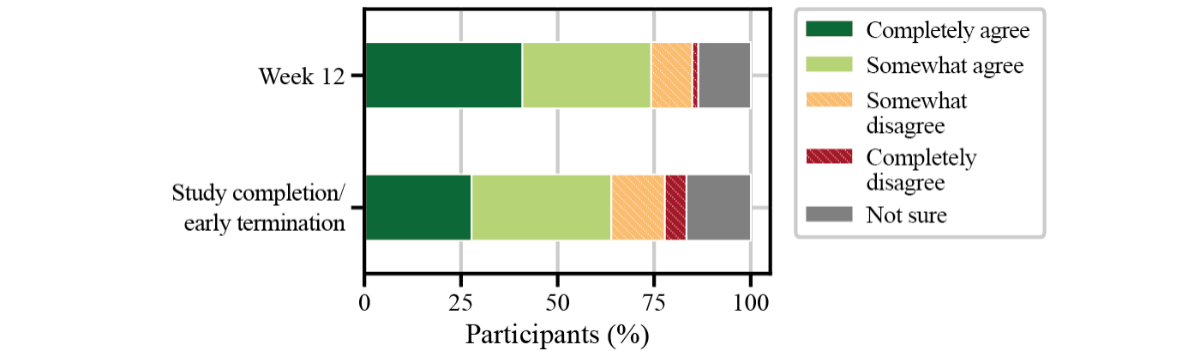
Implications of FLOODLIGHT in people with multiple sclerosis for “desire to continue using the FLOODLIGHT app” from the patient satisfaction questionnaire.

**Figure 10:**
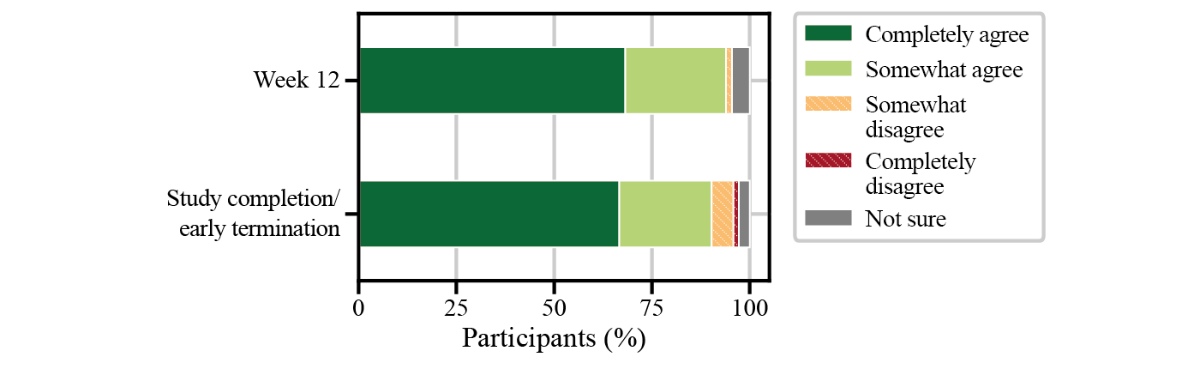
Implications of FLOODLIGHT in people with multiple sclerosis for “prefer to see results immediately to monitor” from the patient satisfaction questionnaire.

Implications for the use of the FLOODLIGHT test battery in people with multiple sclerosis were assessed from individual questions from the patient satisfaction questionnaire. When asked to rate the impact of the smartphone, smartwatch, and active tests on daily living, more than 80% (61/72) of people with multiple sclerosis perceived the FLOODLIGHT test battery to have at least acceptable impact on daily activities (Figure 7). Nearly 50% (32/71) of participants had no issue with any of the active tests, and only one-third would prefer to avoid the 2MWT, most likely due to increased burden from execution—for example, having to find a place to perform the test or not wanting to go outside in bad weather (Figure 8). Without providing any data feedback to the people with multiple sclerosis throughout the study, more than 60% (46/72) of participants would have liked to continue using FLOODLIGHT “to understand my MS better and improve my disease management” (Figure 9). Approximately 90% (65/72) of people with multiple sclerosis indicated their interest to see the results of the tests, which will be addressed in future Roche-sponsored studies using FLOODLIGHT (CONSONANCE [NCT03523858] and FLOODLIGHT Open [floodlightopen.com]; Figure 10). Analysis of patient responses to the satisfaction questionnaire is described in Multimedia Appendix 1.

## Discussion

### Principal Findings

This study demonstrates that the use of smartphones and smartwatches for remote daily active testing and continuous passive monitoring is feasible over 6 months and provides further support to earlier studies, which have shown that healthy participants and people with multiple sclerosis were capable of completing daily tasks on a smartphone [7]. This study provides further evidence for the use of digital technology, including smartphones, for data collection. Other studies in MS have used smartphone apps to (1) assess steps when walking on a treadmill [9]; (2) assess pain, fatigue, anxiety, and quality of life [13]; and (3) assess the feasibility of gathering passive and active performance data [7]. Together with the current analyses, these studies document the focus toward developing digital measures to continuously monitor and assess the MS disease.

In this protocol, the FLOODLIGHT solution collected metrics on cognition, mood, upper extremity function, and gait and posture by instructing participants to perform a set of daily active tests, which should take approximately 5 minutes in total to complete and capture activity data via passive monitoring over a period of 24 weeks. A previous study has shown that 51% of participants (22/38 of people with multiple sclerosis and 17/38 of healthy participants) completed 12 months of daily data collection, where participants were prompted to complete one assigned test [7]. In the context of the FLOODLIGHT study, we observed that overall adherence to active tests was 70% (16.68/24 weeks), which appears to be higher than the adherence of participants to 12 months of daily data collection (39/76, 51%) from Bove et al [7]. However, comparisons between the studies are limited, as the study design and burden of testing are different—for example, the app from Bove et al contained 19 different tests, of which participants were prompted to complete one each day. As the FLOODLIGHT app was integrated into standalone devices in this study, deployment of the app on participants’ own mobile devices may increase adherence because it removes the need to carry a separate, dedicated device and decreases burden on the individual.

### Limitations

As this study remains a pilot investigation designed to collect first experiences from continuous sensor data capture, the main limitation is the small sample size and short duration of follow-up. Future ongoing FLOODLIGHT studies (CONSONANCE and FLOODLIGHT Open) will collect longer term data on smartphone-based sensor data capture in a larger number of participants from a broader disability spectrum. Additionally, whether physical and cognitive limitations in people with secondary progressive MS (SPMS) and people with primary progressive MS (PPMS) differentially impacts adherence compared with people with relapsing MS (RMS) cannot be gleaned from the current data set due to the low numbers of advanced patients enrolled in the study; however, this important question warrants future research exploring remote monitoring in patients with more advanced MS. The importance of continuous monitoring in RMS should also not be overlooked, as the sensitivity of this novel approach aiming at detecting progression in a real-world setting may provide an earlier window into disease progression outside of the clinic.

### Comparison With Prior Work

A recent study assessing the feasibility of the MS TeleCoach, a novel intervention offering telemonitoring of fatigue and telecoaching of physical activity in people with multiple sclerosis, showed that participants were highly engaged, with 76% (57/75) of participants completing the study, and 91% (21/23) of a subset of completers showing a median of quite satisfied in the patient satisfaction questionnaire [27]. During the 12-week study period, use of the MS TeleCoach improved fatigue levels in people with multiple sclerosis with moderate to severe fatigue, suggesting that implementation of digital technologies can enhance patient performance. Together with the data presented here, these results indicate that the use of consumer devices by people with multiple sclerosis for sensor data capture fulfills the prerequisites of people with multiple sclerosis satisfaction and acceptable adherence to daily active tests and passive monitoring for potential integration in long-term clinical trials and treatment monitoring. Regarding future studies, for example FLOODLIGHT Open, attempts will be made to improve participant adherence throughout the study by introducing controlled app variations, such as reminders, types of achievements, and fun metrics.

### Conclusions

In summary, these analyses showed that people with multiple sclerosis are highly engaged with performing active tests and capturing continuous data via passive monitoring and are satisfied with the FLOODLIGHT test battery. Neither satisfaction nor adherence showed strong correlation with study population characteristics. More than 60% (46/72) of people with multiple sclerosis indicated their interest to continue to use FLOODLIGHT, and approximately 90% (65/72) wanted to see the results of their tests in real time as biofeedback, which was implemented in future studies using the FLOODLIGHT solution. These findings indicate that smartphone-based FLOODLIGHT outcomes may represent a promising avenue to enable a more accurate and continuous assessment of MS disease in clinical trials and real-world practice settings and may eventually also contribute to informing and guiding clinical research and clinical practice in the future.

## Appendix

Multimedia Appendix 1 Additional trial information.

Multimedia Appendix 2 Satisfaction questionnaire.

Multimedia Appendix 3 Participant flow table.

## Abbreviations

2MWT: Two-Minute Walk Test
5UTT: 5 U-Turn Test
9HPT: 9-Hole Peg Test
BBS: Berg Balance Scale
DMQ: Daily Mood Question
EDSS: Expanded Disability Status Scale
FAS: full analysis set
FLAIR: fluid-attenuated inversion recovery
FSMC: Fatigue Scale for Motor and Cognitive Functions
HCs: healthy controls
MRI: magnetic resonance imaging
MS: multiple sclerosis
MSIS-29: Multiple Sclerosis Impact Scale–29
MSST: Multiple Sclerosis Symptom Tracker
PDDS: Patient Determined Disease Steps
PHQ-9: Patient Health Questionnaire–9
PPMS: primary progressive multiple sclerosis
PRO: patient-reported outcome
RMS: relapsing multiple sclerosis
RRMS: relapsing-remitting multiple sclerosis
SBT: Static Balance Test
SDMT: Symbol Digit Modalities Test
SPMS: secondary progressive multiple sclerosis
T25FW: Timed 25-Foot Walk
