# Correction: Adherence and Satisfaction of Smartphone- and Smartwatch-Based Remote Active Testing and Passive Monitoring in People With Multiple Sclerosis: Nonrandomized Interventional Feasibility Study

**DOI:** 10.2196/16287

**Published:** 2019-10-08

**Authors:** Luciana Midaglia, Patricia Mulero, Xavier Montalban, Jennifer Graves, Stephen L Hauser, Laura Julian, Michael Baker, Jan Schadrack, Christian Gossens, Alf Scotland, Florian Lipsmeier, Johan van Beek, Corrado Bernasconi, Shibeshih Belachew, Michael Lindemann

**Affiliations:** 1 Department of Neurology-Neuroimmunology Multiple Sclerosis Centre of Catalonia Vall d’Hebron University Hospital Barcelona Spain; 2 Department of Medicine Autonomous University of Barcelona Barcelona Spain; 3 Division of Neurology University of Toronto Toronto, ON Canada; 4 Department of Neurology University of California, San Diego San Diego, CA United States; 5 Department of Neurology University of California, San Francisco San Francisco, CA United States; 6 Genentech Inc South San Francisco, CA United States; 7 F Hoffmann–La Roche Ltd Basel Switzerland; 8 Department of Economics Baden-Wuerttemberg Cooperative State University Loerrach Germany

In “Adherence and Satisfaction of Smartphone- and Smartwatch-Based Remote Active Testing and Passive Monitoring in People With Multiple Sclerosis: Nonrandomized Interventional Feasibility Study” by Midaglia et al (J Med Internet Res 2019;21(8):e14863), there were inconsistencies in the phrases used to describe people with multiple sclerosis. These inconsistencies were inadvertently included during the production process.

The following revisions to the manuscript have been made:

- Throughout the manuscript, all instances of the phrase “persons with multiple sclerosis” have been revised to “people with multiple sclerosis”, including one misspelled instance in the Abstract.
- In the first paragraph of the Introduction, the phrase “people with MS” has been revised to “people with multiple sclerosis”.
- In Table 1, two instances of the acronym “PwMS” within the table have been changed to “people with multiple sclerosis”. Furthermore, the table footnote which explained the acronym has been deleted: “^c^PwMS: people with multiple sclerosis.”
- In Table 2, three instances of the acronym “PwMS” within the table have been changed to “people with multiple sclerosis”. Furthermore, the table caption has been revised from “Demographics and characteristics of people with multiple sclerosis (PwMS) and healthy controls (HC) at baseline” to “Demographics and characteristics of people with multiple sclerosis and healthy controls (HCs) at baseline.”
- The abbreviation list has been updated to remove the term “PwMS: people with multiple sclerosis”.

Furthermore, all instances of “healthy controls” have been correctly abbreviated to “HCs” (whereas the instances in Table 2 and the definition in the Abbreviations list were previously missing the final “s”).

Lastly, the authors wish to correct an error in Table 2 which previously read “relaxing-remitting multiple sclerosis” but has now been changed to “relapsing-remitting multiple sclerosis”.

The changes made do not affect the findings of the study.

The correction will appear in the online version of the paper on the JMIR website on October 8, 2019, together with the publication of this correction notice. Because this was made after submission to PubMed, PubMed Central, and other full-text repositories, the corrected article has also been resubmitted to those repositories.

