# Supplementary material for: Adherence and Satisfaction of Smartphone- and Smartwatch-Based Remote Active Testing and Passive Monitoring in People With Multiple Sclerosis: Nonrandomized Interventional Feasibility Study
Source: J Med Internet Res. 2019 Aug 30;21(8):e14863. doi: 10.2196/14863 (PMC6743265; doi:10.2196/14863)
Supplement: Multimedia Appendix 1 [file jmir_v21i8e14863_app1.pdf]

Multimedia Appendix 1.

Materials and Methods: Trial design and participants

For women of childbearing potential, the eligibility criteria also included the agreement to use an acceptable birth control method during the study period. For HCs, the eligibility criteria were restricted to the ability to comply with the study protocol, age 18–55 years and weight 45–110 kg. Recruitment of HCs was planned to help validate the use of smartphone and sensor-based tests for use in MS versus normative data. Both childbearing potential and weight were restricted in this study as it involved physical tasks.

Materials and Methods: Data transfer

During active tests and passive monitoring, smartphone and smartwatch sensor data were recorded, including accelerometer, gyroscope, compass, Global Positioning System (GPS), ambient light (proximity), Wi-Fi access point signal strength and ID, and cell tower signal strength data. Touch data were recorded during the active tests that require PwMS and HCs to touch the screen.

Participants' data from smartphone- and smartwatch-based assessments were asymmetrically encrypted and uploaded via the internet to a secure central server, maintained by the sponsor, each time the smartphone connected to Wi-Fi. Participants received instructions on how to connect their smartphone to the internet at home. If there was no Wi-Fi available, data were uploaded during the site visits.

Results: Patient satisfaction

Analysis of the association between individual questions from the patient satisfaction questionnaire and PwMS population characteristics indicated that the following questions were significant: "How satisfied or dissatisfied are you with the guidance from the instructor?" correlated with the T25FW test (Spearman's correlation=–0.35, P = .003); "How satisfied or dissatisfied are you with how easy it is to use the smartphone?" correlated with SDMT correct responses (Spearman's correlation=0.25, P = .03); and "How would you rate the frequency with which you were asked to do the active tests?" correlated with the T25FW test (Spearman's correlation=0.34, P = .004).

Analysis of patient responses to the satisfaction questionnaire indicated that the 2MWT was the component of the FLOODLIGHT test that participants would most like to avoid. The analysis of this subgroup of PwMS who would prefer to avoid the 2MWT at study completion identified no significant association of this response with a specific pattern of MS disease or demographic features. The only significant associations were age (Spearman's correlation = –0.34, P = .006) and the T25FW test (Spearman's correlation = –0.26, P = .04), and only at the Week 12 assessment of the patient satisfaction questionnaire, where younger participants were more likely to prefer to avoid the 2MWT in the middle of the study.
