# Supplementary material for: Adherence and Satisfaction of Smartphone- and Smartwatch-Based Remote Active Testing and Passive Monitoring in People With Multiple Sclerosis: Nonrandomized Interventional Feasibility Study
Source: J Med Internet Res. 2019 Aug 30;21(8):e14863. doi: 10.2196/14863 (PMC6743265; doi:10.2196/14863)
Supplement: Multimedia Appendix 2 [file jmir_v21i8e14863_app2.pdf]

Multimedia Appendix 2. Satisfaction questionnaire

Satisfaction questionnaire assessing patients' and healthy volunteers' experience regarding smartphone and smartwatch use and its impact on their daily activities.

Instructions: Please take some time to think about your level of satisfaction or dissatisfaction with the smartphone, the smartwatch and the apps of this clinical study. We are interested in your evaluation of the convenience of the smartphone, the smartwatch and the app over the duration of the study. For each question, please place a single check mark next to the response that most closely corresponds to your own experiences.

1. How satisfied or dissatisfied are you with **how easy it is to use the smartphone**?
    - Extremely Dissatisfied
    - Dissatisfied
    - Somewhat Dissatisfied
    - Neither Satisfied nor Dissatisfied
    - Somewhat Satisfied
    - Satisfied
    - Extremely Satisfied
2. How satisfied or dissatisfied are you with **how easy it is to use the Floodlight App**?
    - Extremely Dissatisfied
    - Dissatisfied
    - Somewhat Dissatisfied
    - Neither Satisfied nor Dissatisfied
    - Somewhat Satisfied
    - Satisfied
    - Extremely Satisfied
3. How satisfied or dissatisfied are you with **how easy it is to put on and take off the smartwatch**?
    - Extremely Dissatisfied
    - Dissatisfied
    - Somewhat Dissatisfied
    - Neither Satisfied nor Dissatisfied
    - Somewhat Satisfied
    - Satisfied
    - Extremely Satisfied
4. How satisfied or dissatisfied are you with **how easy it is to use the smartwatch**?
    - Extremely Dissatisfied
    - Dissatisfied
    - Somewhat Dissatisfied
    - Neither Satisfied nor Dissatisfied
    - Somewhat Satisfied
    - Satisfied

- Extremely Satisfied

5. How **easy or hard is it to plan when you will do your active tests each time**?
    - Very Hard
    - Hard
    - Easy
    - Very Easy
6. How would you rate the frequency with which you were asked to do the active tests?
    - Frequency of tests was unacceptable (too frequent)
    - Frequency of tests was acceptable
7. Taking all things into account, **how satisfied or dissatisfied are you with the smartphone, smartwatch and apps**?
    - Extremely Dissatisfied
    - Very Dissatisfied
    - Somewhat Dissatisfied
    - Neither Satisfied nor Dissatisfied
    - Somewhat Satisfied
    - Very Satisfied
    - Extremely Satisfied
8. To what extent do you agree with the following statement? **I would like to continue using the Floodlight App** to understand my MS better and improve my disease management.
    - Completely agree
    - Somewhat agree
    - Somewhat disagree
    - Completely disagree
    - Not sure
9. To what extent do you agree with the following statement? In future **I would like to be able to see the results of my tests** straight after I've completed them and monitor over time.
    - Completely agree
    - Somewhat agree
    - Somewhat disagree
    - Completely disagree
    - Not sure
10. How satisfied or dissatisfied are you with the **provided information/training materials**?
    - Extremely Dissatisfied
    - Dissatisfied
    - Somewhat Dissatisfied
    - Neither Satisfied nor Dissatisfied
    - Somewhat Satisfied
    - Satisfied

- Extremely Satisfied

11. How satisfied or dissatisfied are you with the **guidance from the instructor**?
    - Extremely Dissatisfied
    - Dissatisfied
    - Somewhat Dissatisfied
    - Neither Satisfied nor Dissatisfied
    - Somewhat Satisfied
    - Satisfied
    - Extremely Satisfied
12. Does the use of smartphone, smartwatch and the active tests **have any impact on your daily activities**?
    - None
    - Minimal
    - Acceptable
    - Substantial
    - Unacceptable
13. If you could **avoid** one of the components of FLOODLIGHT monitoring, which one would that be (check only one)?
    - None
    - Cognitive Test
    - Squeeze a Shape
    - Draw a Shape
    - Balance Test
    - U-Turn Test
    - Two-Minute Walk Test
    - Passive monitoring
14. Additional feedback/comments that you would like to share:
