# Supplementary material for: Adherence and Satisfaction of Smartphone- and Smartwatch-Based Remote Active Testing and Passive Monitoring in People With Multiple Sclerosis: Nonrandomized Interventional Feasibility Study
Source: J Med Internet Res. 2019 Aug 30;21(8):e14863. doi: 10.2196/14863 (PMC6743265; doi:10.2196/14863)
Supplement: Multimedia Appendix 3 [file jmir_v21i8e14863_app3.pdf]

Multimedia Appendix 3. Participants discontinuing from study and excluded from adherence analysis.

Cohort	Participant ID	Reason for withdrawing from study	Excluded from adherence analysis
PwMS	1015	Monitoring frequency-related burden	
PwMS	1019	The participant did not have enough time to implement all study requirements	
HC	1025	Due to personal reasons, the participant was not able to continue with the study	
HC	1035	Lack of availability	X
HC	1036	Lack of availability because of new job	X
HC	1047	Privacy of data. The participant was not comfortable with the fact that the smartphone had GPS	
HC	1052	Participant's personal reasons	
PwMS	1061	Adverse event	X
HC	1062	Lack of availability to come to the visits	X
HC	1076	The participant started to work and did not have availability to come to CEMCAT for the final visit	X
PwMS	2001	Walking task was too exhausting. Concerned about accuracy of data (due to walking task)	
HC	2004	The participant did not want to complete tasks	
PwMS	2006	Travel time to get to study site	
PwMS	2009	Lack of time/interest	
HC	2014	Lives 4 hours away. Was unable to come for last visit	X

Legend: CEMCAT: Multiple Sclerosis Centre of Catalonia; GPS: Global Positioning System; HC: healthy control; PwMS: people with multiple sclerosis.
